# Effects of human and porcine bile on the proteome of *Helicobacter hepaticus*

**DOI:** 10.1186/1477-5956-10-27

**Published:** 2012-04-25

**Authors:** Arinze S Okoli, Mark J Raftery, George L Mendz

**Affiliations:** 1 School of Medical Sciences, The University of New South Wales, New South Wales, Australia; 2 GenØK-Centre for Biosafety, Tromsø Science Park, Tromsø N-9294, Norway; 3 Bioanalytical Mass Spectrometry Facility, The University of New South Wales, New South Wales, Australia; 4 School of Medicine, Sydney, The University of Notre Dame, New South Wales, Australia

**Keywords:** Bile, Bile acids, *Hepaticus*, *Helicobacter*, Proteome, Oxidative stress, Host adaptation, Virulence, Colonization

## Abstract

**Background:**

*Helicobacter hepaticus *colonizes the intestine and liver of mice causing hepatobiliary disorders such as hepatitis and hepatocellular carcinoma, and has also been associated with inflammatory bowel disease in children. In its habitat, *H. hepaticus *must encounter bile which has potent antibacterial properties. To elucidate virulence and host-specific adaptation mechanisms of *H. hepaticus *modulated by human or porcine bile, a proteomic study of its response to the two types of bile was performed employing two-dimensional gel electrophoresis (2-DE) and mass spectrometry.

**Results:**

The 2-DE and mass spectrometry analyses of the proteome revealed that 46 proteins of *H. hepaticus *were differentially expressed in human bile, 18 up-regulated and 28 down-regulated. In the case of porcine bile, 32 proteins were differentially expressed of which 19 were up-regulated, and 13 were down-regulated. Functional classifications revealed that identified proteins participated in various biological functions including stress response, energy metabolism, membrane stability, motility, virulence and colonization. Selected genes were analyzed by RT-PCR to provide internal validation for the proteomic data as well as provide insight into specific expressions of motility, colonization and virulence genes of *H. hepaticus *in response to human or porcine bile.

**Conclusions:**

Overall, the data suggested that bile is an important factor that determines virulence, host adaptation, localization and colonization of specific niches within host environment.

## Introduction

*Helicobacter hepaticus *belongs to the genus *Helicobacter *whose best known specie is the human gastric carcinogen *Helicobacter pylori*. Members of the genus are Gram-negative, microaerophilic bacillar bacteria that are classified either as gastric or enterohepatic species (EHS) depending on their target organs. *H. hepaticus *is the prototype EHS and the most studied species of the group. It was first isolated in 1992 from the liver specimen of laboratory mice suffering from chronic hepatic inflammation and liver cancer [1], and it is now known that the infection caused by the bacterium is widespread among mouse colonies worldwide [2-4]. In humans, *H. hepaticus *has been associated with hepatocellular carcinoma (HCC), cholangiocarcinoma (CC) [5,6], cholelithiasis [7], and inflammatory bowel disease [4,8-10]. Experimental infection with the bacterium has been used as a model of microbial tumor promotion in the liver, colon and mammary glands [11-13], providing ample opportunity to elucidate specific relationships of this group of bacteria with the hosts.

The detection of *H. hepaticus *DNA in human bile samples [9] of biliary disease patients and in specimens of patients suffering from HCC and CC [5], provided links between infection with the bacterium and hepatobiliary diseases in humans. In mice suffering from different hepatobiliary disorders, *H. hepaticus *is routinely cultured from their colon and liver. However, in specimens from patients with various hepatobiliary disorders in which bacterial DNA had been detected, attempts to culture the bacterium have largely been unsuccessful, suggesting that factors in the host hepatobiliary environment play a significant role in determining the adaptability, culturability, virulence and overall physiology of the bacterium.

Bile is an environmental factor present in the colon and hepatobiliary tracts of higher animals with which *H. hepaticus *and other intestinal bacteria must come in contact in their hosts. Bile is a complex dietary secretion composed of bile salts, lipids, proteins, ions, creatinine, pigments such as bilirubin and biliverdin, etc.; accordingly, the response of intestinal flora to bile has multiple facets. Bile salts are the major component of bile and have surface-active amphipathic properties, which can disrupt the lipid bilayer of cell membranes, a property of bile salts that confers antibacterial activity on bile. There are differences in the antibacterial potency of different bile acids: unconjugated bile acids are more toxic than conjugated bile acids, and the bactericidal effect of dihydroxyl bile acids is greater than that of the trihydroxyl bile acids. The composition of bile acids in the bile vary between vertebrate species; under physiological conditions the predominant bile acids in fowl, mice and cattle are chenodeoxycholic, muricholic and cholic acids respectively [14,15]. The composition of bile acids in human bile is made up of *ca*. 40% cholic acid, 40% chenodeoxycholic acid and 20% deoxycholic acid, with traces of ursodeoxycholic acid and lithocholic acid [16]; while the bile acid composition of porcine bile is *ca*. 30% glycocholic acid, 40% taurocholic acid, 7% taurodeoxycholic acid, 15% glycodeoxycholic acid and 5% hyodeoxycholic acid. Porcine and bovine bile are the two commercially available bile types as at the time of this study, and the response of *H. hepaticus *to bovine bile has been investigated [17]. In this study, the global responses of *H. hepaticus *to human and porcine bile were compared with the aim of elucidating specific bacterial-host adaptation and virulence expression mechanisms.

## Results and discussion

### Growth inhibition and morphological changes of *H. hepaticus *ATCC 51449 in the presence of human and porcine bile

**Figure 1:**
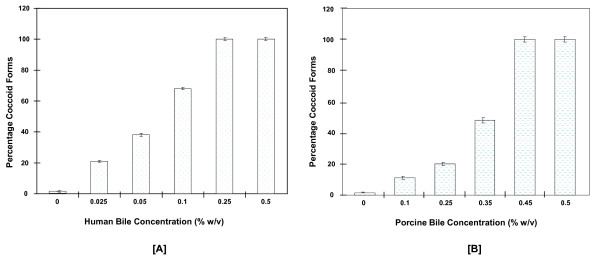
**Change in morphology of *H. hepaticus *in the presence of human bile [A] and porcine bile [B]**. In both types of bile, the percentage of coccoid forms in the bacterial population was higher at high bile concentrations. Data and standard errors are from three independent experiments.

The growth of *H. hepaticus *in human or porcine bile was tested. The bacterium was grown under a microaerobic condition for 48 h in media supplemented with 0-0.5% concentrations of human or porcine bile. Changes in bacterial morphology from normal helical-bacillar to spherical-coccoid cells were observed in both human and porcine bile that were similar to those observed in bovine bile by *H. hepaticus *[17] and *H. pylori *[18]. The morphological changes were observed in greater number of bacteria at lower concentrations of human bile than porcine bile, for example, at 0.1% bile concentration, approximately 68% of the bacterial cell population in human bile had become coccoid compared to the 11% of bacteria in the porcine bile (Figure 1).

**Figure 2:**
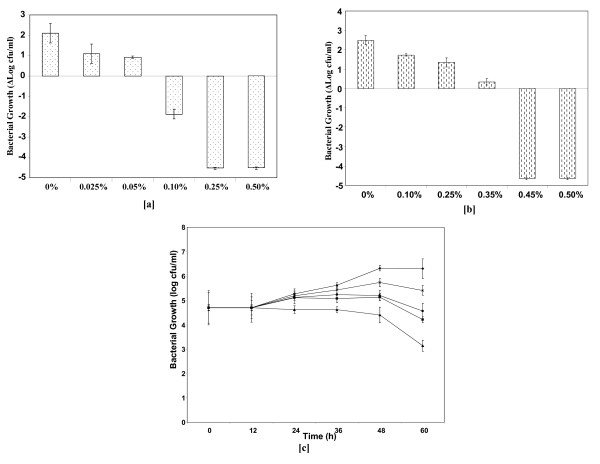
**Growth of *H. hepaticus *in medium containing different concentrations of [a] human bile and [b] porcine bile**. The controls were cultures without bile **[c]**. Growth rate of *H. hepaticus *between 0 and 60 h in 0% bile (♦), 0.05% human bile (■), 0.1% human bile (▲), 0.1% porcine bile (×), and 0.25% porcine bile (●). The data and standard errors are from three independent experiments.

The growth of bacteria depended on the type of bile as well as on bile concentrations (Figure 2). *H. hepaticus *exhibited higher susceptibility to human bile than to porcine bile. At 0.025% human bile concentration, there was a reduction of viable cells by *ca*. 1-log unit representing half of the bacterial density relative to the control cultures without bile (Figure 2a). In contrast, a much higher porcine bile concentration of 0.25% was required to observe a similar *ca*. 1-log reduction in the bacterial viable counts (Figure 2b). This represents a ten-fold higher toxicity of the human bile to *H. hepaticus*. The observed difference in susceptibility could be attributed to differences in the physico-chemical properties of the bile acids present in both types of bile. Human bile is composed predominantly of unconjugated primary bile acids, cholic and chenodeoxycholic acids [19], whereas in porcine bile predominantly conjugated secondary bile acids, taurocholic and glycocholic acids are present. The effects of porcine bile on *H. hepaticus *growth were similar to those of bovine bile [17]: no viable cells were found at approximately 0.5% concentration of both types of bile although the decrease in viability was faster initially in bovine bile. Like porcine bile, bovine bile is also predominantly composed of conjugated secondary bile acids (40% taurocholic acid and 20% glycocholic acid). Mouse is the natural host of *H. hepaticus*, and its bile is made up mostly of muricholic acid, a secondary bile acid, [20]; mouse bile could not be included in this study because of the difficulty in obtaining it in sufficient quantity.

Unconjugated primary bile acids are more toxic than conjugated secondary bile acids because the former can flip-flop passively across the lipid bilayer of cell membranes and enter the cells. In the cells, accumulated primary bile acids dissociate leading to reduction in internal pH and dissipation of the transmembrane proton gradient [21]. Therefore, in addition to their membrane damaging effects, primary bile acids can cause inhibition of cell growth by either intracellular acidification and/or transmembrane proton gradient dissipation. This could account for the greater susceptibility of *H. hepaticus *to human bile than to porcine or bovine bile, and may be a reason for the inability to culture the bacterium from human samples in which its DNA has been detected since the presence of DNA would not be an indicator that culturable bacteria are present. The antibacterial effects of bile could explain also the greater difficulty in culturing *H. hepaticus *from liver than from intestinal specimens [22,23] in mice, since in the former it would be present at higher concentration and unconjugated primary bile acids are found in greater proportion in the liver than in the intestine. Thus, the difference in the composition of bile acids in various organs within the host could determine *H. hepaticus *colonization of its niche as well as viability in laboratory cultures.

Measurement of growth rates of *H. hepaticus *in 0%, 0.05% and 0.1% human bile, and in 0.1% and 0.25% porcine bile showed exponential growth up until 48 h in 0% bile, 0.05% human bile and 0.1% porcine bile concentrations (Figure 2c). Thus, 0% bile (control condition), 0.05% human bile and 0.1% porcine bile (test conditions) were chosen as the conditions to perform the proteomic and transcriptomic studies.

### Identification of proteins differentially expressed by *H. hepaticus *in cultures with sub-lethal human or porcine bile concentrations

**Figure 3:**
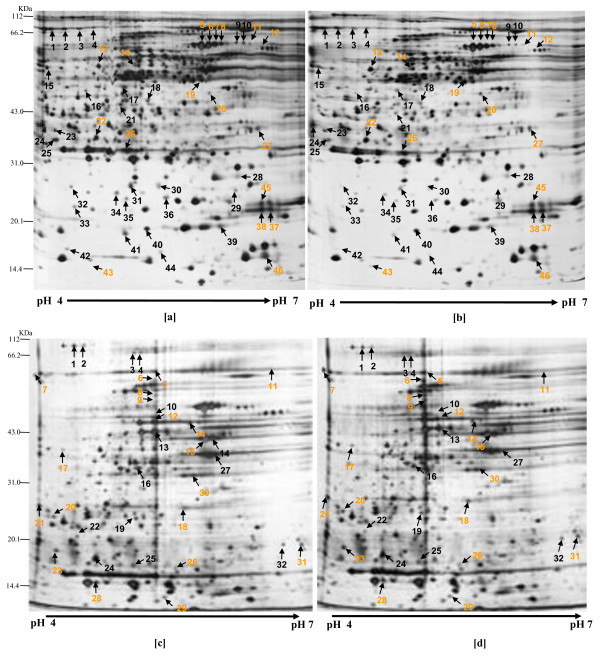
**2D-gels showing *H. hepaticus *cytosolic protein profile at pI 4-7**. Proteins were extracted from bacterial cells grown at 0% Human bile [a], 0.05% Human bile [b]; 0% Porcine bile [c], and 0.1% Porcine bile [d]. Differentially expressed protein spots are numbered on the gels, orange characters indicate up-regulated spots while dark characters indicate down-regulated spots. Proteins in spots were identified by LC-MS/MS and listed in Table 1.

Differential expression of proteins that could play various roles in the ability of *H. hepaticus *to adapt to human or porcine bile was obtained employing 2D-PAGE. Based on the data from previous [17] isoelectric point and grand average hydropathy analyses indicating that the majority of *H. hepaticus *proteins with isoelectric points between 4 and 7 are cytosolic, the present investigation focused on the expression of this set of proteins in the presence of human and porcine bile concentrations of 0.05% and 0.1%, respectively. The protein expression of cells grown in the absence of bile was employed as control and compared to those of cells grown separately in the two types of bile as test cultures. The expression of proteins with differential spot intensities equal to or greater than 2-fold between cells grown in test and control cultures are considered to be modulated by the presence of bile; those protein spots were subsequently analyzed by mass spectrometry after trypsin digestion. Figure 3 shows 2D gels of cell protein extracts of *H. hepaticus *grown in the absence or in the presence of 0.05% human bile or 0.1% porcine bile. Analyses of the proteomes revealed that 46 proteins were differentially expressed in human bile, 18 up-regulated and 28 down-regulated (Figure 3a, b). In cultures with porcine bile, a total of 32 proteins were differentially expressed of which 19 were up-regulated, and 13 were down-regulated (Figure 3b, c).

The identities of the proteins differentially expressed are listed in Table 1. Among these proteins were several potential isoforms; these were proteins identified in more than one protein spot. In the gels from human bile/*H. hepaticus *co-cultures, potential isoforms of dihydropicolinate reductase (DapA), GTP-binding protein (YihK), ferredoxin oxidoreductase (PorA), aconitase (AcnB), and flagella were identified. In the gels from cultures with porcine bile, potential isoforms of ATP-dependent CLP protease (ClpA) and AcnB were identified (Table 1). These findings suggested that various proteins were posttranslationally modified and may manifest adaptations to human and porcine bile stress. Posttranslational protein modification has been reported also in *C. jejuni *and *Wolinella succinogenes *cultures in the presence of bile [24,25].

**Table 1:** Proteins of *H.hepaticus *identified by LC-MS/MS whose expression was modulated by at least 2-fold in 0.05% human- or 0.1% porcine bile in the growth media

**Protein**^**a**^	**Fold change (± SEM)**^**b**^		Gene	**Function**^**c**^	**Spot No**.		**Matching score**^**d**^		Coverage (Peptide)		**Observed/Theoretical pI**^**e**^	
	Human	Porcine			Human	Porcine	Human	Porcine	Human	Porcine	Human	Porcine
**Amino acid metabolism**												
Dihydropicolinate reductase*	0.4 ± 0.01	3.5 ± 0.03	*dapB*	Lysine biosynthesis	33	23	438	201	33(7)	26(12)	6.59/6.24	5.7/6.24
Dihydropicolinate reductase*	0.1 ± 0.01	NI			32	-	430	-	44(19)	-	6.50/6.24	-
Glycine hydroxymethyltransferase	0.48 ± 0.1	2.5 ± 0.01	*glyA*	Glycine hydroxymethyltransferase	18	17	330	278	26(6)	16(5)	6.57/6.33	6.95/6.33
Ketol-acid reductoisomerase	2.7 ± 0.3	NI	*ilvN*	Valine, leucine and isoleucine biosynthesis	27	-	916		49(14)	-	6.59/6.05	-
Threonyl-tRNA synthetase	NI	4.0 ± 0.01	*thrS*	Glycine, threonine and serine metabolism	-	8	-	214	-	12(3)	-	5.78/6.06
2-isopropylmalate synthase	NI	5.0 ± 0.01	*leuA*	Valine, leucine and isoleucine biosynthesis	-	9	-	148	-	26(12)	-	5.78/5.86
Phosphoglycerate dehydrogenase	NI	0.4 ± 0.02	*serA*	Serine family amino acid biosynthesis	-	32	-	583	-	18(9)	-	5.70/5.84
**Cellular metabolism**												
ATP-dependent CLP protease*	NI	0.3 ± 0.01	*clpA*	Endopeptidase activity	-	3	-	941	-	41(19)	-	5.92/5.70
ATP-dependent CLP protease*	NI	0.2 ± 0.06			-	4	-	1167	-	38(36)	-	5.84/5.70
**Carbohydrate & energy metabolism**												
Aconitase*	0.25 ± 0.01	0.4 ± 0.01	*acnB*	Citrate cycle, glyoxylate and dicarboxylate metabolism, reductive carboxylate cycle	2	2	1237	784	48(28)	10(13)	6.65/6.21	6.46/6.21
Aconitase*	0.1 ± 0.03	0.4 ± 0.01			3	1	1491	665	29(20)	29(11)	6.54/6.21	6.54/6.21
Aconitase*	0.1 ± 0.01	NI			1	-	1488	-	43(14)	-	6.76/6.21	-
Aconitase*	0.2 ± 0.01	NI			4	-	866	-	34(12)	-	6.46/6.21	-
Alkyl hydroperoxide reductase	NI	3.0 ± 0.01	*tsaA*	Oxidative phosphorylation	-	28	-	288	-	50(17)	-	6.3/5.95
ATP synthase subunit A	NI	4.0 ± 0.02	*atpA*		-	12	-	891	-	43(19)	-	5.7/5.69
ATP synthase subunit B	NI	2.5 ± 0.1	*atpB*		-	15	-	1509	-	53(12)	-	5.12/5.17
dDTP-glucose dehydratase	0.3 ± 0.01	NI	*rfbB*	Nucleotide sugar biosynthesis	23	-	549	-	31(5)	-	5.57/6.05	-
UDP-glucose 6-dehydrogenase	0.24 ± 0.06	NI	*kfiD*	Pentose and glucuronate interconversions; starch & sucrose metabolism, nucleotide sugars metabolism	17	-	384	-	50(17)	-	5.51/5.27	-
Ferredoxin oxidoreductase*	3.5 ± 0.02	NI	*porA*	Pyruvate metabolism	37	-	514	-	11(4)	-	5.92/5.91	-
Ferredoxin oxidoreductase*	2.5 ± 0.3	NI			22	-	900	-	35(20)	-	6.38/5.91	-
Fumarase	0.25 ± 0.1	0.4 ± 0.1	*fumC*	Citrate cycle, reductive carboxylate cycle	30	13	566	725	47(8)	23(11)	6.62/5.61	5.7/5.61
Fumarate reductase	NI	6.0 ± 0.1	*frdA*	Citrate cycle, oxidative phosphorylation	-	6	-	734	-	38(36)	-	5.7/7.23
Fructose-bisphosphate aldolase	2.8 ± 0.01	NI	*fba*	Glycolysis, gluconogenesis	19	-	975	-	50(13)	-	6.59/5.95	-
Isocitrate dehydrogenase	NI	5.5 ± 0.02	*icd*	Citrate cycle	-	5	-	865	-	23(7)	-	5.7/7.10
Malate dehydrogenase	NI	2.0 ± 0.01	*mdh*	Pyruvate metabolism	-	21	-	710	-	42(9)	-	6.89/8.11
Phosphoglycerate kinase	2.5 ± 0.01	NI	*pgk*	Glycolysis, glyconogenesis	13	-	699	-	44(10)	-	6.0/5.64	-
Rod shape determining protein	2.5 ± 0.2	NI	*mreB*	Cell shape determining protein	20	-	455	-	33(7)	-	5.27/5.16	-
**Cellular oxygen metabolism & stress response**												
Catalase	NI	0.1 ± 0.01	*kat*	Superoxide metabolism	-	10	-	715	-	27(5)	-	5.7/6.75
Superoxide dismutase	0.1 ± 0.01	0.4 ± 0.3	*sodF*		16	27	137	411	13(2)	31(9)	6.49/6.24	5.7/6.24
Thioredoxin reductase	0.3 ± 0.01	4.7 ± 0.03	*trxB1*	Oxygen & ROS metabolism	31	18	470	390	27 (12)	14(5)	5.41/5.34	5.30/5.34
Putative thioredoxin reductase (HH1153)	0.2 ± 0.01	NI	*trxB2*		24	-	381	-	16(5)	-	5.95/6.15	-
**Chaperone & stress response**												
Hsp-70 (DnaK cofactor)	2.3 ± 0.03	NI	*grpE*	Heat shock protein	26	-	308	-	11(8)	-	4.57/4.62	-
Cpn60	NI	3.0 ± 0.01	*groEL*	Chaperonin	-	14	-	705	-	42(11)	-	5.32/5.19
**Lipid metabolism**												
Acetyl-CoA caroxylase alpha subunit	0.2 ± 0.01	NI	*accA*	Fatty acid biosynthesis	41	-	277	-	32(4)	-	5.59/5.47	-
7-Alpha-dehydroxysteroid dehydrogenase	2.5 ± 0.01	NI	*fabG*		46	-	629	-	23(11)	-	6.19/7.59	-
(3R)-hydroxymyristoyl ACP dehydratase	NI	4.0 ± 0.01	*fabZ*		-	7	-	356	-	28(6)	-	6.95/6.31
s-malonyltransferase	0.3 ± 0.01	NI	*fabD*		15	-	311	-	31(5)	-	5.49/5.45	-
**Motility & chemotaxis**												
Flagellin*	2.5 ± 0.3	2.0 ± 0.01	*flaB*	Flagella assembly	11	11	946	961	41(16)	60(11)	4.81/6.38	4.57/6.38
Flagellin*	0.3 ± 0.02	NI			10	-	833	-	13(10)	-	4.95/6.38	-
Flagellin*	0.4 ± 0.01	NI			9	-	699	-	26(9)	-	4.89/6.38	-
Flagellin assembly protein*	0.4 ± 0.01	NI	*fliH*		35	-	474	-	31(5)	-	4.86/4.93	-
Flagellin assembly protein*	0.3 ± 0.01	NI			34	-	466	-	57(8)	-	4.80/4.93	-
Major flagellin subunit	2.4 ± 0.1	NI	*flaA*		12	-	827	-	47(13)	-	4.68/5.56	-
Two-component system response regulator	0.4 ± 0.01	NI	*ompR*	Two-component signal regulatory system	36	-	154	-	16(9)	-	4.84/5.63	-
**Nucleotide metabolism**												
Adenylate kinase	0.2 ± 0.3	NI	*adk*	Purine metabolism	40	-	833	-	35(20)	-	5.22/5.09	-
DNA polymerase III subunit beta	3.0 ± 0.01	NI	*dnaN*	DNA metabolism	45	-	385	-	24(4)	-	5.62/5.33	-
**Pathogenesis & virulence**												
Cytolethal distending toxin	0.1 ± 0.01	NI	*cdtC*	Virulence	44	-	444	-	20(9)	-	4.81/5.23	-
Urease subunit A	NI	0.3 ± 0.03	*ureA*	Urease activity, virulence	-	24	-	393	-	52(8)	-	5.32/5.93
**Protein translation & modification**												
Translation elongation factor Ts	3.2 ± 0.01	2.0 ± 0.1	*ef-ts*	Protein translation	14	30	53	717	16(17)	36(12)	6.0/5.31	5.3/5.31
Translation elongation factor Tu	2.5 ± 0.01	2.0 ± 0.1	*ef-tu*		43	26	64	173	11(6)	15(13)	6.81/4.93	5.49/5.12
**Signal transduction**												
GTP-binding protein*	3.5 ± 0.01	NI	*yihK*	Signal transduction	5	-	948	-	50(12)	-	5.38/5.26	-
GTP-binding protein*	3.0 ± 0.02	NI			6	-	706	-	42(11)	-	5.43/5.26	-
GTP-binding protein*	2.7 ± 0.01	NI			7	-	954	-	31(11)	-	5.49/5.26	-
GTP-binding protein*	3.5 ± 0.01	NI			8	-	638	-	37(9)	-	5.54/5.26	-
Putative ATP/GTP-binding protein	3.5 ± 0.02	NI	*mpr*		38	-	700	-	26(17)	-	6.08/5.77	-
Amino-acid ABC transporter periplasmic solute-binding protein	0.3 ± 0.1	NI	*pbpB*	ABC transporter protein	39	-	396	-	24(4)	-	4.97/5.17	-
Putative ABC transporter protein	0.4 ± 0.01	NI	*yaeC*		29	-	494	-	57(5)	-	4.81/5.16	-
Single-stranded DNA-binding protein	NI	0.3 ± 0.1	*ssb*	Single strand binding protein/primosomal replication	-	25	-	238	-	27(4)	-	5.38/5.91
**Putative protein function**												
HH1212	0.1 ± 0.03	NI	*hh1212*	Hypothetical protein	25	-	349	-	14(5)	-	6.34/6.98	-
HH0952	0.3 ± 0.02	NI	*hh0952*		28	-	267	-	9(3)	-	4.27/4.52	-
HH1514	0.4 ± 0.01	NI	*hh1514*		42	-	286	-	17(9)	-	5.97/5.62	-
HH1023	0.2 ± 0.01	0.2 ± 0.01	*hh1023*		21	16	1312	410	43(21)	31(6)	5.86/6.15	5.86/6.15
HH1098	NI	4.0 ± 0.01	*hh1098*		-	31	-	156	-	10(2)	-	5.7/5.71
HH1376	NI	0.3 ± 0.01	*hh1376*		-	19	-	121	-	11(3)	-	5.89/5.62
HH1034	NI	2.5 ± 0.1	*hh1034*		-	20	-	410	-	53(9)	-	6.73/6.22
HH1426	NI	0.4 ± 0.1	*hh1426*		-	22	-	348	-	28(6)	-	6.49/6.12
HH0243	NI	2.5 ± 0.01	*hh0243*		-	29	-	297	-	22(4)	-	5.59/5.63

^a^Proteins were identified by LC-MS/MS and MASCOT searches of the NCBI nr database. ^b^Proteins that were differentially expressed in the presence of 0.05% human bile or 0.1% porcine bile by at least 2-fold level of expression. A fold change value of > 2 indicates up-regulation, while a value of < 0.5 indicates down-regulation. ^c^Proteins were classified into functional categories based on KEGG pathway classification and DAVID gene ontology, and by comparison with the published literature. ^d^Proteins with peptides with matching scores of > 49 were considered to be identified. ^e^Predicted isoelectric point of each protein was obtained from the Comprehensive Microbial Resource of the Institute of Genomic Research. NI = Not identified.

### Functional classification of identified proteins

Functional analysis of proteins was done with the Kyoto Encyclopedia of Genes and Genomes (KEGG) (http://www.genome.jp/kegg/), the Database for Annotation, Visualization and Integrated Discovery (DAVID 2.1) (http://david.abcc.ncifcrf.gov/), and by comparison with the published literature. The functions of identified proteins are listed on Table 1.

#### Stress response

Evidence for the induction of oxidative stress response in *H. hepaticus *by both human and porcine bile was provided by the down-regulation of the TCA cycle enzymes, AcnB and fumarase (FumC). In particular, the down-regulation by both types of bile of AcnB, which in the TCA cycle is the most sensitive enzyme to reactive oxygen species (ROS) [26], supports this interpretation. Many bactericidal antimicrobial agents are known to stimulate production of hydroxyl radicals, which contribute to cell death via a mechanism which includes hyperactivation of the electron transport chain that stimulates superoxide formation [27]. An increased concentration of this radical negatively affects iron-sulfur clusters leading to the release of ferrous ions which become available for oxidation by the Fenton reaction with the generation of hydroxyl radicals capable of damaging DNA and proteins [27]. The down-regulation of AcnB and FumC may reflect a strategy to counteract a similar toxicity mechanism induced by bile through the reduced production of isocitrate and malate, metabolites that feed electrons to the electron transport chain. As a result, the activities of Fenton reactions will be down-regulated and the generation of superoxide radicals will decrease. The down-regulation of the typical oxygen scavangers, superoxide dismutase and catalase, would reflect lower levels of superoxide anions in the cell. Somewhat counterintuitive was the apparent up-regulation in porcine bile of fumarate reductase, which catalyzes the production of NADH and fumarate from succinate and NAD^+^; increased production of NADH, which is utilized in the electron transport chain would concomitantly lead to increased ROS production. Perhaps this unexpected up-regulation of fumarate reductase can arise from the bacterial response that may be multiplexed, such as a strategy to up-regulate its anaerobic respiration, in which the enzyme catalyzes the final step of ATP synthesis with fumarate as the terminal electron acceptor [28]. This strategy would reduce the levels of hydroxyl radicals generated from superoxide anions produced in aerobic respiration by increasing anaerobic respiration in preference to aerobic respiration. In response to bovine bile [17], down-regulation of fumarate reductase together with other enzymes of TCA suggested a strategy by the bacterium to reduce succinate and other metabolites utilized in the electron transport chain in order to minimize the effects of superoxide radicals.

Likewise, the up-regulation of pyruvate:ferredoxin reductase (PorA) in the presence of human bile could show a strategy by the bacterium to minimize generation of ROS. PorA is a known NADPH:paraquat diaphorase (NADPH:PQ^2+^), and participates in the control of singlet oxygen production through the reduction of PQ^2+ ^in the cell [29]. The down-regulation of acetyl-CoA carboxylase and S-malonyltransferase, both of which consecutively catalyze the conversion of Ac-CoA to carboxyl carrier proteins, suggested the preservation of Ac-CoA by the cells. This would preserve levels of the coenzyme for the synthesis of citrate under conditions in which the TCA enzymes are down-regulated. Maintenance of adequate intracellular levels of citrate may be required under conditions in which the TCA cycle is down-regulated because it is a strong chelator of ferric ions; the available citrate would contribute to the sequestration of iron and thus to the reduction of hydroxyl radical production. A similar control of hydroxyl radical levels was used in the response of *H. hepaticus *to bovine bile [17], suggesting that decreasing ROS production is a response mechanism common to different types of bile.

In the presence of porcine bile, protection against hydroxyl radicals would be helped by the up-regulation of TrxB1 and alkyl hydroperoxide reductase (TsaA). Up-regulation of isocitrate dehydrogenase (Icd), which catalyzes the oxidative decarboxylation of isocitrate to α-ketoglutarate with concomitant production of NADH could increase ROS production, but this can be compensated by α-ketoglutarate, which interacts with oxygenases to reduce molecular oxygen. In contrast, the presence of human bile induced a down-regulation of the thioredoxin reductases as well as a down-regulation of the *tsaA *transcript, although TsaA was not found among the regulated proteins of *H. hepaticus *in response to human bile; this would hinder the ability of the bacterial cells grown in human bile to control ROS. Up-regulation of NAD(P) + -dependent oxidoreductase 7-alpha-hydroxysteroid dehydrogenase (FabG) would add to the production of NADH or NADPH for cellular biosynthetic reactions that may be required to compensate for the down-regulation of the TCA cycle, but this could result also in increased generation of hydroxyl radicals. FabG was not identified among the regulated proteins of *H. hepaticus *cells exposed to porcine bile. Together, these observations suggested that larger amounts of hydroxyl radicals would be generated in the bacterial responses to human bile than to porcine bile, and this will help to account for the higher susceptibility of *H. hepaticus *to the former.

FabG was up-regulated in the presence of human bile, in contrast to its down-regulation in cultures with bovine bile [17] and non-regulation by porcine bile; these differences may reflect an attempt to detoxify the cholic and chenodeoxycholic acids found in higher proportions in human bile than in porcine and bovine bile. Hydroxylation renders bile more hydrophilic and consequently less toxic [30]; and 7-dehydroxylation by the enzyme FabG is restricted to few anaerobic intestinal bacteria which represent a small fraction of the total colonic flora [19]; modulation of this enzyme by *H. hepaticus *in the presence of bile could be a mechanism that the bacterium and possibly other EHS use in their colonization of the enterohepatic habitat [31]. The enzyme has been associated with tumour progression in susceptible mice [32] and may contribute to tumour formation.

The up-regulation of the chaperones GrpE and GroEL in cultures with human and porcine bile, respectively, indicated an increased need by *H. hepaticus *to maintain proper folding of polypeptides in order to counteract the damaging effects of bile acids on proteins and cell membranes. In addition, *H. hepaticus *appeared to respond to bile by undergoing important changes involving DNA synthesis and repair, as well as protein translation and transcription. This was manifested in the up-regulation of DNA polymerase III subunit beta in human bile, and would serve to improve error free DNA repair during DNA replication, as well as in the up-regulation by both types of bile of the elongation factors EF-Ts and EF-Tu, that participate in protein translation, stress response and DNA repair [33,34]. Up-regulation of EF-Ts and EF-Tu has also been reported in the bile response of other Campylobacterales [24,25], suggesting common bile adaptation mechanisms of this bacterial group.

#### Energy balance

To survive the effects of bile requires energy, for example to extrude bile acids out of the cytoplasm and to maintain proper protein folding and homeostasis. The down-regulation of TCA cycle reactions and decreased electron supply to the electron transport chain by *H. hepaticus *in the presence of bile would lower the energy available for bile resistance. In response to porcine bile, up-regulation of Icd and the ATP synthase subunits A and B would serve to provide additional energy to the bacterial cells. In contrast, ATP synthase was not found among the regulated proteins of *H. hepaticus *in response to human bile; also, the up-regulated phosphoglycerate kinase and fructose-bisphosphate aldolase involved in glycolysis cannot translate into increased production of energy as *H. hepaticus *lacks pyruvate kinase to catalyze the substrate-level phosphorylation that uses ADP and phosphoenolpyruvate as substrates downstream of glycolysis [35]. The lowered expression of the ATP-dependent ABC transporters PbpB and YaeC reflected the lower energy in the bacterium as a consequence of the effects of human bile and were consistent with the relative growth data in porcine and human bile.

#### Outer membrane stability

Dihydropicolinate reductase (DapB) catalyzes the fourth step in the biosynthesis of meso-diaminopimelate, an essential intermediate for the synthesis of bacterial peptidoglycan. The up-regulation of this enzyme suggested the triggering of enhanced repair of damaged cell-wall components by the bacterium in response to porcine bile. This is consistent with the up-regulation also of 2-isopropylmalate synthase, an enzyme involved in the synthesis of hydrophobic amino acids [36] required for the repair of damaged membrane components and stabilization of protein-membrane associations. In response to human bile, ketol-acid reductoisomerase, involved in the synthesis of hydrophobic amino acids [36], and the cell shape protein, MreB involved in the co-ordination of cell morphogenesis [37] were up-regulated. DapB, dDTP-glucose dehydratase and UDP-glucose 6-dehydrogenase will result in decreased synthesis of peptidoglycan as well as dTDP glucose and UDP-D glucuronate, the down-regulation of these enzymes would result in lower levels of these sugars used in cell wall biosynthesis [38] and may compromise the membrane stability of the bacterium. These molecular events would be reflected in a greater susceptibility of *H. hepaticus *to human bile than to porcine bile.

#### Glycine biosynthesis

A physiological role of glycine hydroxymethyltransferase (GlyA) is the reversible interconversion of serine and glycine. It plays a major role in the generation of folate coenzymes, which provide one-carbon units for the biosynthesis of a variety of products such as DNA, RNA, ubiquinone, and methionine [39]. Thus, the down-regulation of GlyA in the presence of human bile and its up-regulation in the presence of porcine bile will affect differently the biosynthesis of these metabolic products by the bacterium in the presence of the two types of bile. Interestingly, phosphoglycerate dehydrogenase (SerA) that catalyzes the conversion of serine to glycerate which eventually enters the glyoxylate pathway was down-regulated by *H. hepaticus *grown in porcine bile and not regulated by human bile. A tendency to conserve serine is a possible strategy to ensure sufficient supply of this amino acid for the synthesis of glycine.

#### Motility

**Figure 4:**
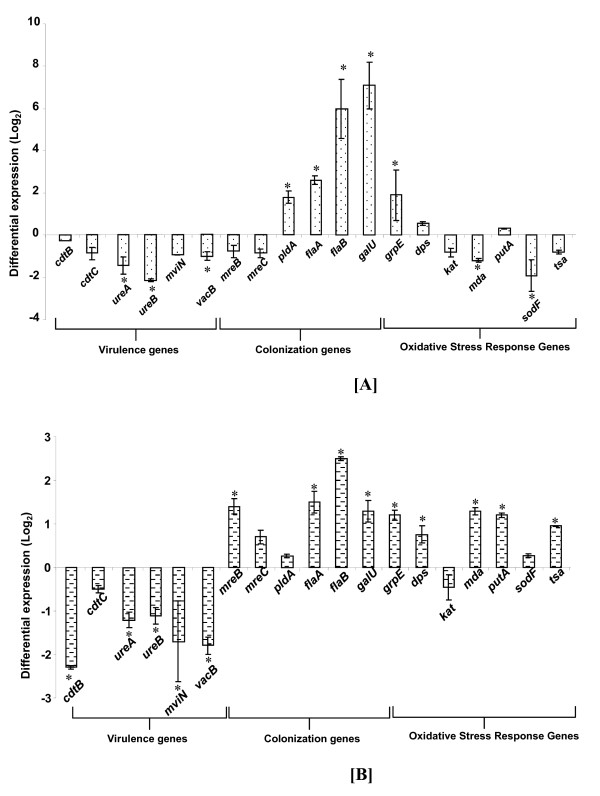
**Differential expression of *Helicobacter hepaticus *virulence, colonization and oxidative stress genes in [A] 0.05% human bile, and [B] 0.1% porcine bile**. 16 S rDNA was used as internal control to normalize the relative expressions of the genes. *H. hepaticus *specific primer set used for the qRT-PCR has been published previously [17] Experiments were performed in triplicate. Data and standard errors are from two independent experiments.

Up-regulation by *H. hepaticus *of the flagellar proteins FlaAB in the presence of human or porcine bile would increase its motility in growth media containing either type of bile. The statistically significant up-regulation (p < 0.05) of the *flaAB *transcripts measured in bacterial cells grown in either type of bile confirmed the observation with the flagellar proteins (Figure 4), and thus served as an internal validation for the proteomic data. It correlated also with the observed speeds of the bacterium in 0.05% and 0.01% human and porcine bile, respectively, which move significantly greater than in the absence of bile (p < 0.01); there was no difference in the percentage of motile cells between the 0.01% and 0.05% human and porcine bile, respectively. Bacterial flagella contain a specialized secretion apparatus that functions to deliver the protein subunits that form the filament and other structures outside the membrane; the flagellar assembly protein FliH regulates the flagellar ATPase FliI activity, preventing non-productive ATP hydrolysis, and also acts to target FliI to the C ring complex of the flagellar basal body [40]. Low ATP levels in *H. hepaticus *grown in the presence of human bile could make the regulatory action of FliH on FliI less important and lead to a decrease in the expression of FliH. As a consequence, the contribution of this protein to the export of FliI will be diminished too. This would not necessarily compromise the export of the flagellar apparatus, since *H. hepaticus *flagellar secretion may require a proton motive force and not ATP hydrolysis by FliI as in the case of *Salmonella enterica *[41]. Down-regulation of the two-component system response regulator OmpR is consistent with the repellant effect of bile on *H. hepaticus*. This agrees with the report that human bile as well as taurocholic and taurodeoxycholic acids (the predominant bile acids found in porcine bile) exerted chemorepellant effects on *H. pylori *[42].

#### Virulence and colonization

Cytolethal distending toxin Cdt and urease are important factors in *H. hepaticus *cytotoxicity, colonization and virulence. Cdt is essential for persistent infection of the gastrointestinal tract and increases the severity of mucosal inflammation or liver disease in susceptible mouse strains [43]. The protein is composed of three subunits: the catalytic subunit CdtB has DNase 1-like activity and targets eukaryotic DNA, and CdtAC are binding proteins for delivering CdtB into target cells. Mutations in CdtABC that cause any of the three subunits to lose function prevent the bacterial cell from inducing cytotoxicity [44]. Urease supports *H. pylori *colonization of the stomach by increasing the pH of the bacterial microenvironment through the hydrolysis of urea [45]. Urease levels of expression in *H. hepaticus *are lower than in *H. pylori*, but relatively high compared with those of many other ureolytic pathogens. Cytolethal distending toxin subunit C (CdtC) and urease subunit A (UreA) were down-regulated by human and porcine bile, respectively. The correlation of these results with the down-regulation of the expressions of *ureA *in the presence of porcine bile as well as of *cdtBC *in the presence of human and porcine bile, although only small down-regulations were measured for *cdtB *in human bile and for *cdtC *in both bile types (Figure 4), served to validate the proteomic data. In porcine bile the bacterium also down-regulated the expression levels of *ureB *and *cdtB*, and in human bile *ureAB *were down-regulated as well. It is noteworthy that in bovine bile *H. hepaticus *also down-regulated CdtC and UreAB [17] indicating that the presence of bile did not increase the pathogenicity of *H. hepaticus *dependent on these factors.

Thus, the results showed that at the physiologically neutral environment of *H. hepaticus *bacterial cultures, the presence of bile down-regulated the expression of *ureAB*, and attenuated the pathogenicity of the bacterium dependent on CdtBC. Down-regulation of virulence factors by bile have been reported also in other bacteria [46,47]. For example, in the presence of bile the expression of the *Salmonella *invasion protein and the Type III secretion proteins are down-regulated; [46] in *Vibrio cholerae*, the production of cholera-toxin and toxin-regulated pilus are significantly down-regulated; [47] and in *C. jejuni*, GalU is down-regulated [24].

### Transcriptional regulation of virulence, colonization and oxidative stress-related genes by *H. hepaticus *in response to bile

Together with the down-regulation of the expressions of *cdtBC *and *ureAB *in the presence of human and porcine bile, the transcript levels of other virulence genes of *H. hepaticus *such as *mviN*, and *vacB *were also down-regulated in cells exposed to both types of bile, although only a small down-regulation was measured in the case of *mviN *for cells exposed to human bile (Figure 4). On the other hand, colonization genes of *H. hepaticus*, namely *flaAB *and *galU*, were up-regulated in both types of bile. The gene *pldA *encoding a phospholipase was significantly up-regulated in human bile but not in porcine bile, while *mreB *showed significant down-regulation only in porcine bile. *H. hepaticus *in response to human bile down-regulated the oxidative stress response genes *kat, mda, sodF *and *tsa*, while the bacterium up-regulated same genes in porcine bile with the exception of *kat *(Figure 4). In the human bile, down-regulation of *sodF *mRNA transcript was in agreement with its down-regulation at the proteome level. Similarly, up-regulation of *tsa *in porcine bile corresponded with the protein up-regulation, however, the up-regulation of *sodF *transcript in the same bile, albeit a non-significant up-regulation (P > 0.05), did not correspond with the down-regulation of the protein. Incongruence between parallel transcriptomic and proteomic experiments has also been reported by others [48,49]. The level of any protein will be the balance between its rate of synthesis and its rate of degradation; thus, a decrease in the level of a protein could be the result of decreased synthesis or enhanced degradation. Since the transcriptional up-regulation of *sodF *in porcine bile could not explain the down-regulation of the protein, it could be hypothesized that decreased turnover rate was responsible for the observed SodF level [50].

Owing to their importance in survival and pathogenesis, the transcriptional regulation of the genes presented in Figure 4 has been investigated in the response to bile of other enteric pathogenic bacteria [51]. The MivN homolog (HH0516) is required by *S. typhimurium, E. coli *and *Burkhoderia pseudomallei *for virulence [52]; the gene *hh0516 *encodes a homologue of the mouse virulence factor of these bacteria. In *B. pseudomallei*, expression of *mivN *is influenced by free-iron availability in the media and by the growth phase [52]. The down-regulation of *mivN *by *H. hepaticus *suggested reduced intracellular free iron concentration, which could result from iron chelation by bile components [53]. The vacuolating cytotoxin B VacB, is required for *H. hepaticus *virulence as well as in *Shigella *and enteroinvasive *E. coli *[54]; *vacB *was down-regulated in the presence of either type of bile. The down-regulation of these genes suggests that bile is not an environmental cue required for *H. hepaticus *virulence.

Up-regulation of the transcript levels of *H. hepaticus *colonization genes *flaAB, galU *and *pldA *suggested that bile can aid in bacterial colonization of its specific niches in the host. Up-regulated *flaAB *would help *H. hepaticus *to move away from bile as discussed previously, but it also can assist the bacterium to reach its niches. Up-regulation of the phospholipase encoded by *pldA *in the presence of bile suggested that like in *H. pylori *where the gene aids the bacterium to colonize the gastric mucosa [55], PldA may be a colonization factor in *H. hepaticus *stimulated by bile. Similarly to *Streptococcus pneumoniae *and *V. cholerae *where *galU *mutants showed reduced ability to colonize the mouse small intestine [56], up-regulation of *galU *by *H. hepaticus *in the presence of either type of bile would suggest that it acts also as a colonization factor activated by bile; it is noteworthy that in *H. hepaticus *response to bovine bile [17], elevated level of GalU was also measured. The up-regulation of the oxidative stress response genes in porcine bile but not in human bile underscores the difference in the pattern of *H. hepaticus *stress responses induced by the presence of human or porcine bile as discussed previously.

## Conclusion

The protein and gene regulations of *H. hepaticus *in response to human and porcine bile observed in this study, as well as to bovine bile [17], provided molecular explanations for the observed susceptibility of the bacterium to the three types of bile (Figure 1) and [17]. *H. hepaticus *adaptation to bile is dependent on the type of bile as well as on bile concentration; in this study the bacterium was found to be more susceptible to human bile than porcine bile. To combat oxidative stress induced by the different bile acids present in human or porcine bile, *H. hepaticus *employed different mechanisms involving modulation of metabolic processes and energy balance. Overall, this ability was more impaired in human bile than in porcine bile; extrusion of bile acids and maintenance of membrane homeostasis in the bacterium appeared to be more compromised in human bile than porcine bile. The expression and/or activities of several proteins involved in virulence, colonization, genetic and physiological interactions of *H. hepaticus *with bile were modulated differently by the bacterium in response to human or porcine bile revealing that bile can be an important factor that determines host adaptation, localization and colonization of specific niches within the host environment.

## Materials and methods

### Bile extracts

Dried unfractionated porcine bile (Sigma, North Ryde, NSW, Australia) was dissolved in Brain Heart Infusion (BHI) broth (Oxoid, Heidelbeg, VIC, Australia) to the appropriate concentrations and filter-sterilized using 0.22 μm filters (Millipore, Kilsyth, Camperdown, VIC, Australia). Human bile was collected from patients at Royal Prince Alfred Hospital, NSW, Australia. The collection was performed under the ethics approval protocol number X05-0343 of 30 May, 2006 titled "Molecular and cellular pathogenesis of human liver disease" granted by the Human Research Ethics Committee (HRECS) of Australia. Bile from different patients were pooled and boiled under atmospheric pressure for 1 h, and vacuum dried for 48 h. The dried bile was then dissolved in BHI broth (Oxoid, Australia) to the appropriate concentrations and filter-sterilized.

### Bacterial strain and growth conditions

*Helicobacter hepaticus *strain ATCC 51449 was cultivated in *Campylobacter *Selective Agar (CSA), consisting of Blood Agar Base No. 2 (Oxoid, Australia), and supplemented with 5% (v/v) defibrinated horse blood (Oxoid, Australia). The media contained 2 μg/ml fungizone (Bristol-Myers Squibb, Sydney, NSW, Australia), 0.32 μg/ml polymixin B, 5 μg/ml trimethoprim, and 10 μg/ml vancomycin (Sigma, Australia). Cultures were incubated for 48 h at 37°C under the microaerobic conditions of 5% CO_2_, 5% O_2 _and 90% N_2_. Bacteria were harvested from plates, washed in BHI broth, resuspended to a density of OD_600 _~0.1. Cell purity was checked by inspecting the characteristics of bacterial colonies on CSA plates and the morphology of bacteria under phase-contrast microsopy. The responses of *H. hepaticus *to human or porcine bile were determined by inoculating bacterial cells at a density of c*a*. 10^4 ^cfu/mL into 2 mL of BHI broth containing 0-0.5% human or porcine bile concentrations in a 24-well microtitre plate. To determine the specific effects of each bile on *H. hepaticus*, bacterial growth was determined as a function of time for various concentrations of human and porcine bile.

### Bacterial motility and morphology in cultures with human or porcine bile

*H. hepaticus *motility and morphological characteristics were determined employing a previously described method [17]. Briefly, at 48 h the percentage of motile bacillar-shaped cells in cultures with 0%, 0.05% and 0.1%, human or porcine bile was evaluated on a scale from 0% (no motile cells) to 100% (all cells in motion). The average time taken by 10 cells to move across the microscope field was recorded and used to compute the average speed of the bacterium. Changes in morphology of *H. hepaticus *in human and porcine bile were observed by phase-contrast microscopy. The percentage of coccoid forms at 48 h in 0-0.5% human or porcine bile at 48 h was estimated by evaluating the number of nonbacillar forms per field in three independent samples of each culture.

### Preparation of cell-free protein extracts

For protein expression studies, cells harvested at exponential growth phase in control cultures or in the presence of 0.05% or 0.1% (w/v) human and porcine bile, respectively were treated with chloramphenicol (128 mg/ml) to terminate protein synthesis. Bacteria in triplicate cultures from at least two independent experiments were centrifuged at 5000 × g at 4 C for 25 min, and the cell pellets were resuspended and washed twice with 0.2 M ice-cold sucrose. The bacteria were then disrupted by thrice freezing and thawing, and the lysate was suspended in 0.5 ml of Tris-SDS-Urea buffer [50 mM Tris, pH 8.0; 0.1% sodium dodecyl sulphate (SDS); 2.5 M urea]. Cell debris was removed by centrifugation at 14000 × g at 4 C for 20 min, and cell-free extracts used for 2-dimensional gel electrophoresis. The protein content of cell-free extracts was estimated by the bicinchoninic acid assay employing a microtitre protocol (Pierce, Rockford, IL, USA); optical densities were measured at 595 nm using a Beckman Du 7500 spectrophotometer to measure the absorbances of the copper complexes in both samples and standards. The protein concentration of each sample was calculated based on a calibration curve constructed with known concentrations of bovine serum albumin (Sigma, Australia).

### Two-dimensional polyacrylamide gel electrophoresis and image analysis

Two-dimensional polyacrylamide gel electrophoresis (2D-PAGE) was performed as described previously [14,15]. Briefly, an 18-cm pH 4-7 immobilized pH gradient (IPG) strip (Amersham Biosciences, Melbourne, VIC, Australia) was rehydrated overnight with 500 μl of isoelectric focusing rehydration buffer that contained 150 μg of the protein extract, 8 M urea, 100 mM dithiothreitol (DTT), 40 mM Tris-HCl pH 8.8, 2% IPG buffer, 65 mM 3-[(3-cholamidopropyl)-dimethylammonio]-1-propanesulfonate and 2% nuclease buffer. Isoelectric focusing was conducted using the Multiphor II system (Amersham Biosciences, Australia) at a maximum of 3500 V for 21 h until a total of 65 500 V-hour were achieved. For the second dimension, strips were equilibrated by rocking in two buffers containing 6 M urea, 20% (v/v) glycerol, 2% (w/v) SDS, 375 mM Tris-HCl; the first buffer with 130 mM DTT, and the second with 135 mM iodoacetamide (IA). Sodium dodecyl sulphate-polyacrylamide gel electrophoresis (SDS-PAGE) was performed on 11.5% acrylamide gels using a Protean II system (Bio-Rad; Regents Park, NSW, Australia) at 50 V for 1 h, followed by 64 mA for 5 h or until the bromophenol blue dye front reached the bottom of the gels. The gels were fixed individually in 200 ml of fixing solution 50% (v/v) methanol, 10% (v/v) acetic acid with gentle rocking for a minimum of 0.5 h, stained employing a silver staining method [57], and imaged using a Umax PowerLook-1000 flatbed scanner (FujiFilm; Tokyo, Japan). For comparative gel-image analysis, statistical data were acquired and analyzed using the Z3 software package (Compugen; Jamesburg, NJ, USA). For analyses, one gel from cells grown in the absence of bile served as the reference gel; master gels were compiled from the three gels from each growth condition, and were compared to determine the relative intensities of each protein spot. Statistical analyses (Student *t*-test, 95% confidence interval) were performed on three gels from each growth condition to determine the differential spot intensities between control and test conditions.

### Identification of proteins

Protein spots showing 2-fold or more differences in intensity between both experimental conditions were identified by liquid chromatography-tandem mass spectrometry (LC-MS/MS) as described before [17]. Briefly, spots were excised from the gels and washed twice for 10 min in 200 μl of 100 mM NH_4_HCO_3_, reduced at 37°C for 1 h with 50 μl of 10 mM DTT, alkylated for 1 h in 50 μl of 10 mM IA, washed for 10 min with 200 μl of 10 mM NH_4_HCO_3_, dehydrated in acetonitrile, and trypsin-digested with 10 ng/μl of trypsin (Promega, Annandale, NSW, Australia). After digestion for 14 h at 37°C, peptides were extracted by washing the gel slice for 15 min with 25 μl 1% formic acid, followed by dehydration in acetonitrile. Digests were then dried *in vacuo*, resuspended in 10 μl 1% formic acid and submitted for Quadrupole-TOF (Q-TOF) analysis on a Micromass instrument which generated CID as previously described [17]. Database searches with Mascot search engine (Matrix Science Ltd.; Boston, MA) was performed as previously described [14,15]. Proteins were identified with high confidence according to a matching score > 49 with more than 2 assigned peptides both corresponding to P < 0.05. All searches were performed on the National Center for Biotechnology Information non-redundant (NCBI nr) database.

### RNA extraction and cDNA synthesis

Total RNA was extracted from bacterial cells suspended in liquid cultures with RNA-protect using the Qiagen method according to the manufacturer's instructions (Qiagen Pty. Ltd.; Doncaster, VIC, Australia) as previously described [17]. RNA samples were stored at -80°C until used. Total RNA was reverse-transcribed to cDNA using the SuperScript III reverse transcriptase following the manufacturer's instructions (Invitrogen; Mulgrave, Victoria, Australia).

### Real Time RT-PCR

To serve as validation for the proteomic data as well as provide insight into the regulation by *H. hepaticus *of virulence, colonization and oxidative stress genes, the effects of human and porcine bile on the transcriptional regulation of selected 19 genes related to virulence, colonization and oxidative stress response were studied. Four independent real-time quantitative PCR experiments were carried out. The analysis of each gene was performed in triplicate for each experiment in a Corbett Research Rotor Gene RG-3000 thermal cycler (Corbett Life Science; Sydney, NSW, Australia), using the SYBR GreenER qPCR Universal Supermix according to the manufacturer's instructions (Invitrogen, Australia). Each reaction was performed in an individual tube in a 72 tube strip, containing 12.5 μl Supermix, 1.0 μl of 100 ng/μl forward primer, 1.0 μl of 100 ng/μl reverse primer, 1.0 μl of 200 ng/μl cDNA, and diethylpyrocarbonate-treated water to a total volume of 25 μl. As controls, reactions were run for each set in the absence of template cDNA to detect any contamination. Conditions for the qRT-PCR were as described previously [17]. The relative expression of each target gene was normalized to the 16 S rDNA gene using the method described by Pfaffl *et al. *[58] employing the expression:

$$\text{Ratio }=\;{\left({\text{E}}_{\text{Target}}\right)}^{\Delta \text{CtTarget}\left(\text{Control}-\text{Sample}\right)}/{\left({\text{E}}_{\text{Reference}}\right)}^{\Delta \text{CtReference}\left(\text{Control}-\text{Sample}\right).}$$

The amplification efficiencies of target and reference genes, E was assumed in this study to be 2 for all genes [59], and Ct is the comparative threshold cycle. The control/sample values were obtained with template cDNA from *H. hepaticus *cells exposed to human bile (0 and 0.05%), and porcine bile (0 and 0.1%). *H. hepaticus *specific primer set used for the qRT-PCR has been published previously [17].

## Competing interests

The authors declare that they have no competing interests.

## Authors' contributions

ASO designed the experiments, analyzed and interpreted the data, and drafted the manuscript. MJR participated in the analysis of the mass spectrometry data. GLM conceived the study, participated in the data interpretation and revised the manuscript. All authors read and approved the final manuscript.
